# 2-Amino-*N*-Phenethylbenzamides for Irritable Bowel Syndrome Treatment

**DOI:** 10.3390/molecules29143375

**Published:** 2024-07-18

**Authors:** Miglena Milusheva, Mihaela Stoyanova, Vera Gledacheva, Iliyana Stefanova, Mina Todorova, Mina Pencheva, Kirila Stojnova, Slava Tsoneva, Paraskev Nedialkov, Stoyanka Nikolova

**Affiliations:** 1 Department of Organic Chemistry, Faculty of Chemistry, University of Plovdiv, 4000 Plovdiv, Bulgaria or miglena.milusheva@mu-plovdiv.bg (M.M.); stoyanova@uni-plovdiv.bg (M.S.); minatodorova@uni-plovdiv.bg (M.T.); 2 Department of Bioorganic Chemistry, Faculty of Pharmacy, Medical University of Plovdiv, 4002 Plovdiv, Bulgaria; 3 Department of Medical Physics and Biophysics, Faculty of Pharmacy, Medical University of Plovdiv, 4002 Plovdiv, Bulgaria; vera.gledacheva@mu-plovdiv.bg (V.G.); iliyana.stefanova@mu-plovdiv.bg (I.S.); mina.pencheva@mu-plovdiv.bg (M.P.); 4 Department of General and Inorganic Chemistry with Methodology of Chemistry Education, Faculty of Chemistry, University of Plovdiv, 4000 Plovdiv, Bulgaria; stoynova@uni-plovdiv.bg; 5 Department of Analytical Chemistry and Computer Chemistry, University of Plovdiv, 4000 Plovdiv, Bulgaria; slava.tsoneva@uni-plovdiv.bg; 6 Department of Pharmacognosy, Faculty of Pharmacy, Medical University of Sofia, 1000 Sofia, Bulgaria; pnedialkov@pharmfac.mu-sofia.bg

**Keywords:** synthesis, mebeverine, spasmolytic activity, anti-inflammatory activity, denaturation of albumin

## Abstract

Irritable bowel syndrome (IBS) is a common gastrointestinal (GI) disorder characterized by abdominal pain or discomfort. Mebeverine is an antispasmodic that has been widely used in clinical practice to relieve the symptoms of IBS. However, its systemic use usually leads to side effects. Therefore, the current paper aimed to synthesize more effective medicines for IBS treatment. We used ring opening of isatoic anhydride for the synthesis in reaction with 2-phenylethylamine. In silico simulation predicted spasmolytic activity for 2-amino-N-phenethylbenzamides. The newly synthesized compounds demonstrated a relaxation effect similar to mebeverine but did not affect the serotonin or Ca^2+^-dependent signaling pathway of contractile activity (CA) in contrast. Having in mind the anti-inflammatory potential of antispasmodics, the synthesized molecules were tested in vitro and ex vivo for their anti-inflammatory effects. Four of the newly synthesized compounds demonstrated very good activity by preventing albumin denaturation compared to anti-inflammatory drugs/agents well-established in medicinal practice. The newly synthesized compounds also inhibited the expression of interleukin-1β and stimulated the expression of neuronal nitric oxide synthase (nNOS), and, consequently, nitric oxide (NO) synthesis by neurons of the myenteric plexus. This characterizes the newly synthesized compounds as biologically active relaxants, offering a cleaner and more precise application in pharmacological practice, thereby enhancing their potential therapeutic value.

## 1. Introduction

Irritable bowel syndrome (IBS) is a common gastrointestinal (GI) disorder characterized by abdominal pain or discomfort and alterations in bowel habits without structural and biochemical abnormalities [1]. IBS produces quantifiable suffering. The quality of life, interpersonal connections, social lives, everyday activities, and productivity at work of individuals with the disorder are all negatively impacted by IBS [2]. Despite the establishment of multiple diagnostic criteria over time, there is no gold standard for the diagnosis of IBS [3,4,5,6]. Currently, the Rome IV criteria are advised for diagnosing the illness [7].

There is usually a positive correlation between the number of extraintestinal symptoms and the severity of IBS [8]. Ninety-five percent of the body’s serotonin (5-HT), which is released as an essential first step in the commencement of gut motility and plays a major role in the general functioning of the intestine, is found in the GI mucosa [9]. The main therapeutic drugs now used to treat IBS work upon the serotonin system, and there is evidence that the disturbance of serotonin signaling in the GI tract has a pathophysiological role in IBS and other GI illnesses. 5-HT is a common connection in various critical GI tract activities, including intestinal secretion, GI motility, and pain perception, among the many neurotransmitters involved in brain–gut axis communication [10,11,12]. Extrinsic afferent nerves originate bowel-related feelings, like bloating and pain, whereas intrinsic enteric nervous system neurons initiate motor and secretory reflexes like peristalsis [13,14,15,16]. 5-HT activates the 5-HT3 receptors on the parasympathetic ganglia, which in turn stimulate the release of acetylcholine (ACh) from nerve terminals, causing smooth muscle (SM) contraction and an increase in intestinal secretions.

Pharmacotherapy mostly focuses on managing symptoms and has a limited impact on IBS [17]. Many of the available medicines are typically not accepted by patients or medical payers [18]. Despite newly developed drugs targeting receptors, some of which are already in use, antispasmodic therapy remains a powerful therapeutic choice for IBS [19].

Mebeverine is an antispasmodic that has been widely used in clinical practice to relieve symptoms of IBS such as abdominal pain and cramps, bloating and flatulence, diarrhea, or constipation [20]. This anticholinergic agent causes normalized intestinal motility by promoting musculotropic and spasmolytic action on the GI smooth musculature. Its mechanisms of action include a reduction in permeability of the ionic channels, a local anesthetic effect, and water absorption alternation [21]. Through these mechanisms, mebeverine provides antispasmodic effects without the permanent relaxation of SM cells in the GI tract known as hypotension [22].

However, its systemic use usually leads to side effects such as skin reactions, nausea, dizziness, and headaches. Adverse effects include hypersensitivity, allergic reactions, immune system disorders, and skin disorders including hives, edema, and widespread rashes. Additionally, the list of reported adverse effects also includes heartburn, indigestion, tiredness, diarrhea, constipation, loss of appetite, general malaise, dizziness, insomnia, headache, and a decreased pulse rate [21].

Since many IBS patients do not respond well to current medical treatments, more effective drug development is needed. Therefore, the current paper aims to synthesize more effective medicine for IBS treatment. In this context, we are interested in finding a new approach to obtaining mebeverine derivatives and establishing a structure–activity correlation. The software-predicted biological activity of newly synthesized molecules is confirmed or rejected by in vitro or ex vivo tests [23,24]. In this way, it is possible to investigate the pharmacological activity and obtain data on the mechanisms of action as well as the duration of the pharmacological effects.

## 2. Results and Discussion

### 2.1. Synthesis of 2-Amino-N-Phenethylbenzamides

**Scheme 1:**
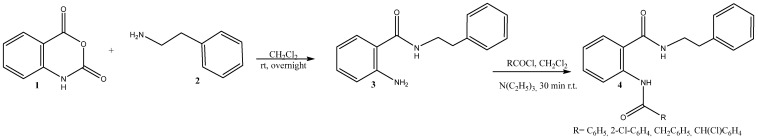
Synthesis of the 2-amino-N-phenethylbenzamides **3** and **4a**–**d**.

Our synthetic methodology was based on the previously described reaction [25,26,27] of isatoic anhydride **1** with 2-phenylethylamine **2** to form the hybrid molecule **3** with high purity and yield (97%), according to Scheme 1. The structure of the resultant molecule was confirmed by FT-IR, ^1^H-NMR, ^13^C-NMR, and MS-spectra, the results of which were completely consistent with data found in the literature [25,26,27].

Next, we turned our attention to the synthesis of amides. The amide group’s existence in biological systems and its critical role in medicinal chemistry make amide synthesis incredibly significant [28,29,30].

Using the amine **3**, we conducted an acylation process to obtain diamides **4**. The acylation was effective, furnishing diamides **4a**–**d** with a 79–81% yield (Table 1).

The structures of all the compounds were verified by spectral data (Supplementary Materials, Figures S1–S12) and were completely consistent with the corresponding molecule and data in the literature for **4b** [31].

### 2.2. In Silico Predictions

Using in silico methods, the drug-likeness of the compounds and their ADMET properties were also predicted theoretically. Increased lipophilicity, membrane permeability, and pharmacological activity are the outcomes of conformational restriction of small drug molecules brought about by the incorporation of this noncovalent interaction into drug design [32].

According to Lipinski’s rule of five [33], the ADME investigation demonstrated that the compounds have good GI absorption and can pass across the blood–brain barrier. We found that **4a**–**d** met the standard values (Table 2) of molecular weight (MW < 400), XLOGP3 ≤ 5.2, ESOL or estimated solubility (log S: not more than 6), polarity (TPSA: 20 to 130 Å^2^), and flexibility (RB: no more than 9), etc.

The substances had a bioavailability score of 0.55, indicating good oral absorption [33]. Additionally, they scored well on the synthetic accessibility scale (1.45–3.11), a crucial factor in drug discovery.

Cytochrome P450 (CYP) inhibition results in pharmacological toxicity [34,35]. The examined substances were not anticipated to function as P-glycoprotein substrates. According to the calculations, every compound should have inhibited the CYP1A2, CYP2C19, CYP2C9, CYP2D6, and CYP3A4 isoform; the only exception being **4d**, which does not inhibit CYP1A2, and CYP2C9 and CYP3A4. All the compounds exhibited good skin permeability, with log Kp ranging from −5.98 to −4.94.

Based on the calculated results, each compound was assumed to be a potential therapeutic candidate.

According to Pro Tox-II acute toxicity data, all the substances had a toxicity class of 4 or 5 (on a 1–6 scale, where 1 is the highest and 6 is the lowest toxicity). The compounds’ estimated LD_50_ was in the range of 2000. The compounds had an estimated LD_50_ of 2000–2025, except for **3** (LD_50_ = 1000 mg/kg) and **4c** (LD_50_ = 600 mg/kg).

After calculating the low toxicity level of the synthesized 2-amino-N-phenethylbenzamine **3** and its amides **4,** we focused on investigating their biological activities. The free software tool PASS Online predicted spasmolytic and anti-inflammatory activity for the synthesized compounds.

### 2.3. Biological Evaluation of 2-Amino-N-Phenethylbenzamides

#### 2.3.1. Spasmolytic Activity

The ex vivo study of the CA of SM tissues, combined with chemical, immunohistochemical, and pharmacological methods and approaches, is a promising way to obtain objective information about the specificities of the biologically active capacity of the new substance being studied.

SMs cannot be voluntarily controlled. Their contraction is regulated by hormones, nerve stimulation from the autonomic nervous system, and local factors [36]. Activation of the SM contraction mechanism is a consequence of changes in the permeability of surface ion channels on the sarcolemma, or a change in the state of the large number of receptors expressed on its surface [37]. It is characteristic of this type of experimental model that the described processes can modulate the concentration of cytosolic Ca^2+^ and, therefore, the reactivity of SM [38] at a low concentration of the investigated substances (up to 10^−9^ M) [39]. This is an exceptional advantage in the experimental determination of the biological activity of newly synthesized molecules.

In this context, the structure–activity relationship determined by a computer program led to the design of mebeverine derivatives, the synthesis and study of which are presented in the current work. This research aims to synthesize mebeverine derivatives and determine their ex vivo and in vitro biological activity.

SMs are found in various systems and organs, providing mechanical stability and the regulation of organ size. SM tissue forms the walls of the airways and blood vessels, and also covers hollow organs such as the bladder, uterus, stomach, and intestines. SMs have the properties of excitability, conduction, and contractility, which are preserved even when they are isolated from the body, due to their autonomous innervation. This characteristic allows them to be used as a basis for SM experimental models capable of simulating and mimicking physiological and pharmacological functions not only in vivo and in situ conditions, but also in in vitro and ex vivo experiments. Using an ex vivo SM model system allows for the study of experimental activity from a biomechanical perspective.

Supported by the structure–tissue activity relationship information arising from empirical screening, the unified structural model of SM activation/inactivation promises to both accelerate drug discovery in this field and improve our fundamental understanding of structure-based new drug design in general.

In this regard, we investigated a chemo-mechanical coupled new substance–contractile isolated tissue model that described the contractile behavior of SM. It is based on a molecule concentration-force function and an active calcium-driven part related to the chemical contraction of SM cells.

**Figure 1:**
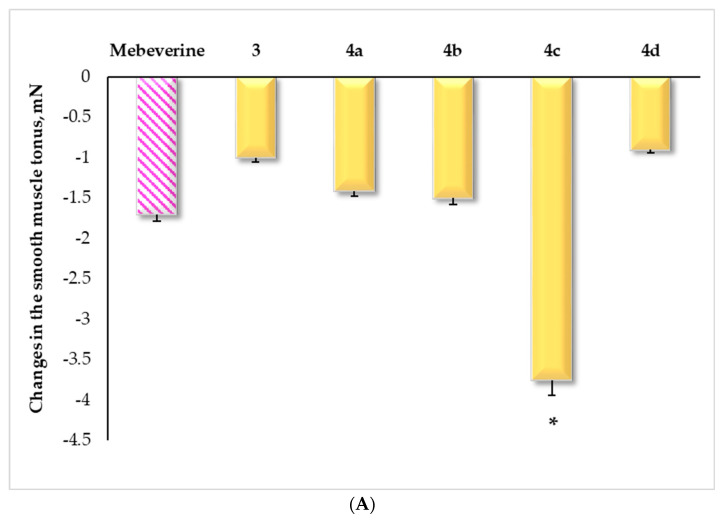

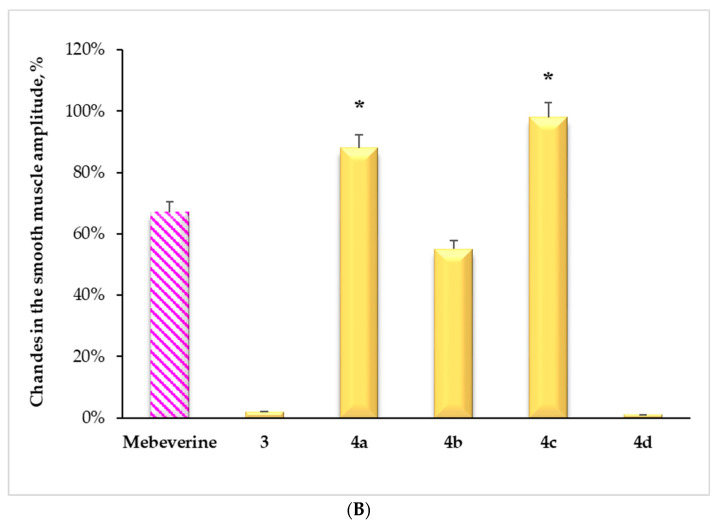
Changes in SM tissue parameters: (**A**) tone in mN; and (**B**) amplitude in %, induced by compounds **3** and **4a**–**d** (5 × 10^−5^ M) (*n* = 12). Statistically significant differences: * *p* < 0.05 comparison is between mebeverine and **3**, **4a**–**d**.

In tissue bath conditions, we observed the main parameters of CA in excitable tissues such as the amplitude and frequency of phasic contraction and the strength of tonic response. For this purpose, initially, SM contractile responses were recorded both in the presence and absence of the neurotransmitter ACh (in concentration 10^−6^ M). This was followed by a 20 min incubation of the isolated SM strips with mebeverine compared to the tested molecules **3**, **4a**–**d**. All substances showed specific activity manifested in changes in the mechanical properties of the muscles and the duration of the initial expression of the pharmacological effect in the concentration range of 10^−4^ M to 10^−6^ M (Figure 1). Mebeverine, applied to the tissue bath at a submaximal concentration of 5 × 10^−5^ M, produced a significant relaxation, reducing the strength of spontaneous contraction tone by 1.7 mN, decreasing the amplitude by 66,7%, and changing the spontaneous contraction frequency by 0.8%. The nature of this medicinal effect was similar for substances **3**, **4a**–**d** when used at the same concentration of 5 × 10^−5^ M, although specific differences were expected. The most significant changes in the main parameters of biomechanical contractions were observed with substances **4a** and **4c**. The impact of **4b** was similar to that of mebeverine, and no significant change in the function of the SM preparations (SMP) was observed after the administration of **4d** (Figure 1).

**Figure 2:**
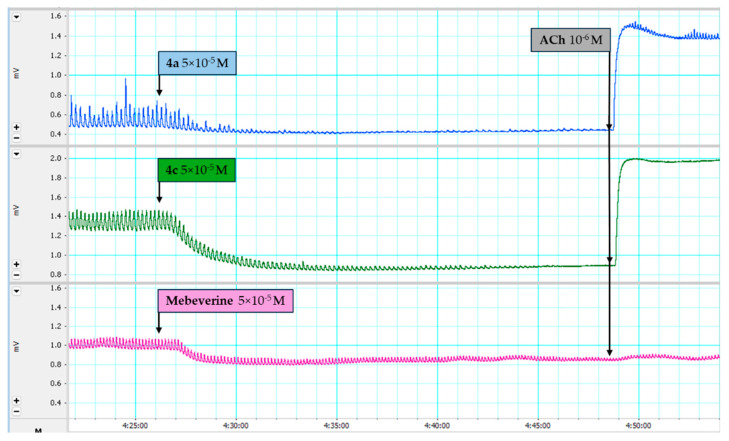
Representative tracings demonstrating the contractile responses of SMPs from the gastric corpus of rats following the application of mebeverine compared to the application of substances **4a** and **4c**, subsequently followed by an ACh reaction in their background.

We found that the most significant difference between mebeverine and the compounds **3**, **4a**–**d,** was in affecting the ACh-mediated pathway of contractile development SM function (Figure 2). We found that all the newly synthesized derivatives **3**, **4a**–**d** preserved the ACh reaction (change from 0.5 to 1.4%); however, after applying mebeverine (in concentration 5 × 10^−5^ M), the ACh reaction was completely reduced (change 99.3%). Interestingly, mebeverine has been used in clinical practice for years for IBS treatment as a direct SM relaxant [33,40] but the activity we found indicated a significant side effect of this drug. Mebeverine blocks an essential neurotransmitter for the entire body and is a prerequisite for affecting the chemical signaling system of intercellular communication [41]. In contrast to this side effect, the substances **3** and **4a**–**d** demonstrated a relaxation effect similar to that of mebeverine but did not affect the main neurotransmitter or the subsequent Ca^2+^-dependent signaling pathway of CA (Figure 2) [42]. This characterizes the newly synthesized compounds as biologically active relaxants, offering a cleaner and more precise application in pharmacological practice, thereby emphasizing their potential therapeutic value.

#### 2.3.2. Anti-Inflammatory Activity

##### In Vitro Inhibition of Albumin Denaturation

The synthesized molecules **3**, **4a**–**d** were tested in vitro and ex vivo for their anti-inflammatory effects. Inflammation is a reaction to an injury or disease. The ability of a medication or therapy to decrease swelling or inflammation is known as its anti-inflammatory activity. Anti-inflammatory medications lessen inflammation and discomfort. To treat inflammation, the following two types of pharmaceuticals are commonly used: non-steroidal anti-inflammatory drugs and steroids. Many negative side effects are associated with non-steroidal medications, including GI issues that may cause stomach ulcers [43].

**Figure 3:**
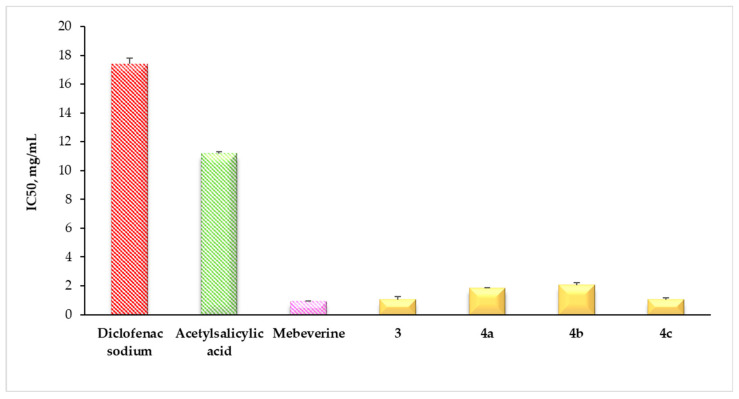
IC_50_ inhibition of albumin denaturation (in mg/mL) of newly synthesized hybrids (yellow). Diclofenac (red) and acetylsalicylic acid (green) were used as standards. The lower the value, the higher the anti-inflammatory activity.

Diclofenac and acetylsalicylic acid, as known anti-inflammatory drugs, were compared to the hybrids **3**, **4a**–**d** to assess their ability to prevent contractions and inflammation in the GI tract. We measured the resistance of albumin’s denaturation by applying the newly synthesized compounds, compared to that of mebeverine and the anti-inflammatory drugs diclofenac and acetylsalicylic acid (Figure 3). Four of the newly synthesized 2-amino-N-phenethylbenzamine **3** and 2-amino-*N*-phenethylbenzamides **4a**–**c** demonstrated very good anti-inflammatory activity in avoiding albumin denaturation compared to acetylsalicylic acid and diclofenac sodium.

Amine **3** (1.05 mg/mL) and amide **4c** (1.04 mg/mL) had similar IC_50_ to mebeverine (0.91 mg/mL), while **4a** and **4b** had higher IC_50_ than mebeverine but demonstrated better activity than the well-known drugs acetylsalicylic acid and diclofenac. **4d** did not show any anti-inflammatory activity.

Further ex vivo experiments were carried out to verify these findings and assess the impact of the hybrids on nNOS and interleukin-1 (IL-1) expression. We carried out additional ex vivo tests to assess the hybrids’ impact on IL-1 expression to establish the presence of an anti-inflammatory effect and an nNOS effect.

#### 2.3.3. Ex Vivo Immunohistochemical Analysis

Nitric oxide (NO) is a simple and distinctive molecule that performs numerous functions in the human body, including acting as an intracellular second messenger and an intracellular mediator [44]. NO exerts a powerful vasodilation effect and affects all metabolic processes [45]. Doubtless, NO plays a major role in controlling blood pressure, as well as playing an active role in immune defense, the operation of cardiovascular, neurological and digestive systems, brain functions (learning and memory), cell lipolysis promotion, etc. [46,47]. NO is produced by nNOS and could be considered as a neurotransmitter, stimulating the NO-sensitive guanylate cyclase and therefore reducing the tone of various types of SMs, including those in blood vessels [48,49,50].

Inflammation plays an important role in the development of atherosclerosis, but the specific stimuli that trigger cytokine release in atherogenesis are unknown. Increased blood pressure may be a cause of inflammation, which is a possible mechanism underlying the well-established role of hypertension as a risk factor for atherosclerotic disease [51]. The well-known mediators of chronic inflammation are pro-inflammatory cytokines such as IL-1α, IL-1β, IL-6, and tumor necrosis factor-α (TNF-α) [52]. Both IL-1β and IL-6 are potent proinflammatory cytokines that act on endothelial cells and SM cells [53]. Other mediators are cyclooxygenase-2, prostaglandin-E2, chemokines (proteins, lipids, etc.), and NO in its three isoforms nNOS, inducible NOS (iNOS), and endothelial NOS (eNOS) [48]. In this respect, a study of new molecules stimulating the expression of NOS1 or NOS3, which would cause an increased local NO production, is especially important. It is crucial to consider the significant clinical and therapeutical implications of NO when developing novel immunomodulating treatments that depend on NOS expression.

**Figure 4:**
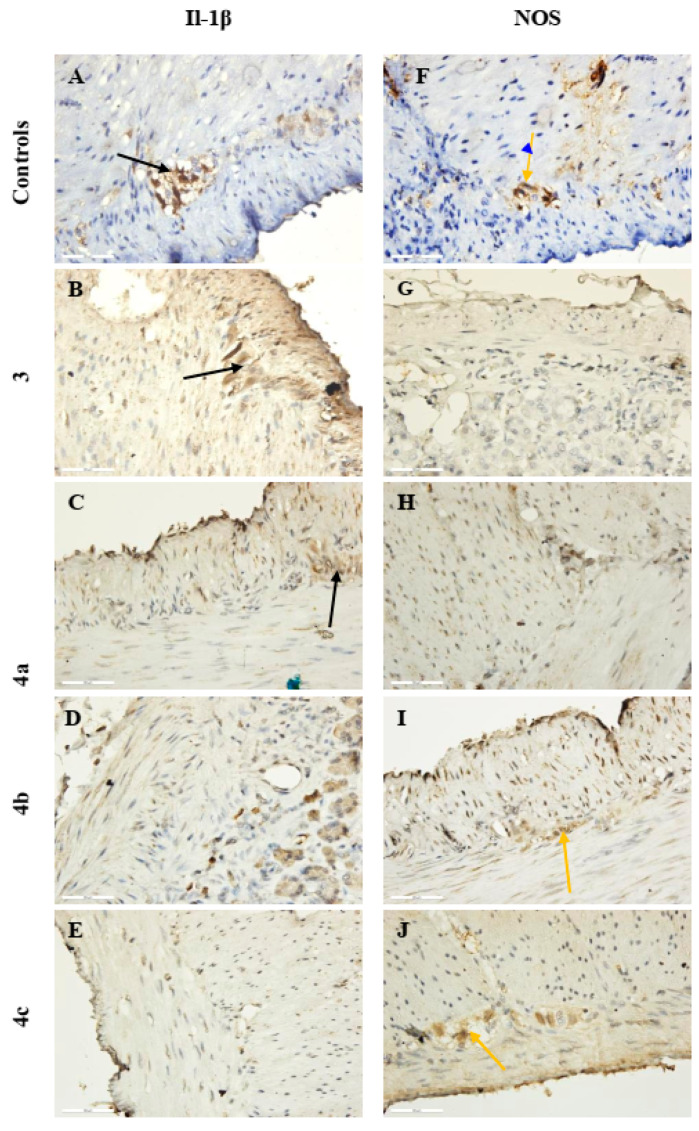
Micrographs of SM stomach corpus preparations with newly synthesized compounds (the incubation period was 3 h): (**A**) control, IL-1β expression (black arrow), at ×400; (**B**,**C**) SMPs incubated with substance **3**, **4a** expression of IL-1β (black arrow), at ×400; (**D**) SMPs incubated with substance **4c**, lack of IL-1β expression in myenteric plexus, weak in mucosal glands, at ×400; (**E**) SMPs incubated with substance **4b**, lack of IL-1β expression, at ×400; (**F**) control, nNOS expression (orange arrow) at ×400; (**G**,**H**) SMPs incubated with substances **3** and **4a**, lack of nNOS expression, at ×400; and (**I**,**J**) SMPs incubated with substances **4b** and **4c**, increased expression of nNOS in the myenteric plexus (orange arrow), at ×400.

We found that **4c** and **4d** promoted/induced anti-inflammatory activity because they suppressed the expression of IL-1β in both SM cells and neurons in the myenteric plexus but stimulated nNOS expression in the SMs and myenteric plexus. On the other hand, **3** and **4a** had no anti-inflammatory potential because they stimulated IL-1β expression and suppressed nNOS expression (Figure 4).

The obtained data were consistent with studies on the role of nNOS in vascular tone regulation. The newly synthesized substances **4b** and **4c** suppressed the expression of IL-1β but stimulated the expression of nNOS in nitrergic neurons in the myenteric plexus.

**Figure 5:**
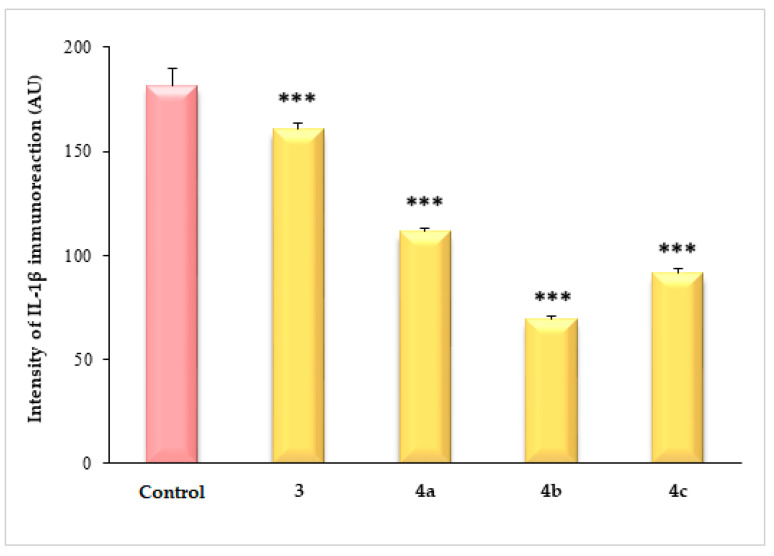
Expression of IL-1β in the SMPs incubated with the newly synthesized compounds **3** and **4.** The asterisk indicates significant differences between groups—*** *p* < 0.001.

The statistical analysis performed revealed a significant change in the intensity of immunoreaction for IL-1β and nNOS in myenteric plexus neurons. The measured mean values for IL-1β in the control (181.8 ± 8.4 AU) showed a statistical difference compared to those treated with substance **3** (160.6 ± 3.2 AU, *p* < 0.001) and substance **4a** (111.6 ± 1.6 AU, *p* < 0.001). The intensity of the immunoreaction was significantly reduced after treatment with substance **4c** (91.49 ± 2.2 AU, *p* < 0.001), but the greatest decrease was observed after treatment with **4b** (69.16 ± 2.2 AU, *p* < 0.001) (Figure 5).

IL-1β expression in the SMPs incubated with the newly synthesized compounds **3**, **4a**, and **4c** indicated a significant decrease in the intensity of the immunoreaction. The lowest values were observed for **4b**. The results showed that all the tested substances have anti-inflammatory properties, but the strongest effect was registered for compounds **4c** and **4b**. As documented in the literature, compounds that suppress the expression of IL-1β induce indirect antioxidant effects by inhibiting inflammation-related oxidative stress, fibrosis, and apoptosis signals [54].

Limited production or bioavailability of NO, on the other hand, is associated with the constriction of blood vessels, leading to increased blood pressure and hypertension, and, consequently, to vascular hypertrophy and stenosis [55]. Therefore, low levels of NO can be associated with various thrombotic processes.

**Figure 6:**
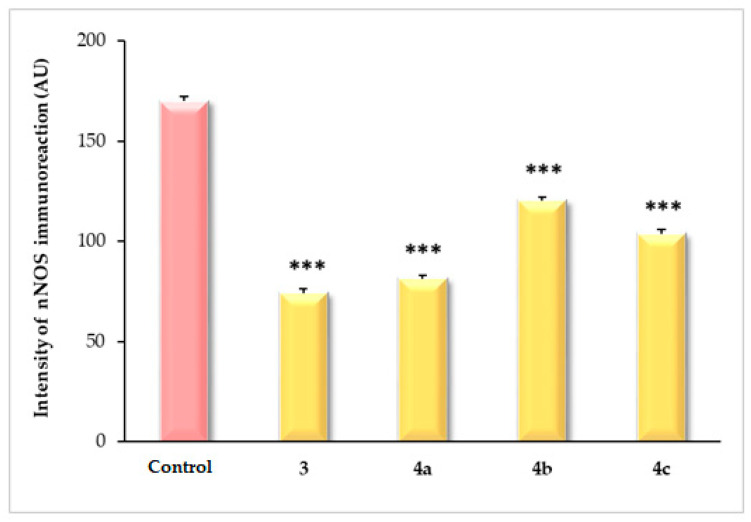
Expression of nNOS in the SMPs incubated with the newly synthesized compounds **3** and **4**. The asterisk indicates significant differences between groups—*** *p* < 0.001.

Comparing the intensity of the immune response for nNOS in control preparations (169.9 ± 2.2 AU, *p* < 0.001) versus those treated with the newly synthesized compounds. we found that the lowest values were observed in preparations treated with substance **3** (74.08 ± 2.2 AU, *p* < 0.001), followed by those treated with **4a** (81.35 ± 1.7 AU, *p* < 0.001) and **4c** (103.8 ± 1.8 AU, *p* < 0.001); the highest values were observed with **4b** (120.4 ± 1.8 AU, *p* < 0.001) (Figure 6).

SMPs incubated with compounds **3**, **4a**–**c**, on the other hand, showed a moderate expression of nNOS. We found that **4b** and **4c** had higher immunoreaction intensities. **4d** did not affect IL-1β or nNOS expression. These results confirm that NO produced by nNOS in neurons can be considered a neurotransmitter that stimulates NO-sensitive guanylyl cyclase in effector cells, thereby reducing the tone of various types of SMs, including stomach and blood vessels [48,49,50].

The obtained data are consistent with studies on the role of nNOS and IL-1β in the regulation of SMs and vascular tone. The newly synthesized compounds **3**, **4a**–**c** inhibit the expression of IL-1β and stimulate the expression of nNOS, and, consequently, the synthesis of NO by neurons of the myenteric plexus.

## 3. Materials and Methods

All solvents and reagents were purchased from Aldrich (Merck, Sofia, Bulgaria, EAD). Melting points were measured on a Kruss M5000 melting point meter (A. Krüss Optronic GmbH, Hamburg, Germany). The purity of the compounds was determined by TLC (precoated 0.2 mm Merck silica gel 60 plates (Merck KgaA, Darmstadt, Germany)). The compounds were characterized by their IR, ^1^H-NMR, ^13^C-NMR, and HRESIMS spectra. NMR spectra were recorded at room temperature (ac. 295 K) on a Bruker Avance III HD 500 spectrometer (Bruker, Billerica, MA, USA) at 500 MHz. HRESIMS spectra were acquired in positive mode on a Q Exactive Plus (Thermo Fisher Scientific, Inc., Bremen, Germany) mass spectrometer, equipped with a heated HESI-II source. Operating conditions for the HESI source used in a positive ionization mode were as follows: +3.5 kV spray voltage, 320 °C capillary and probe heater temperature, sheath gas flow rate 36 a.u., auxiliary gas flow rate 11 a.u., spare gas flow rate 1 a.u. (a.u. refers to arbitrary values set by the Exactive Tune software v.2.4) and S-Lens RF level 50.00. Nitrogen was used for sample nebulization and collision gas in the HCD cell. The aliquots of 1 µL of the solutions of the samples (ca. 20 µg mL^−1^) were introduced into the mass spectrometer through the LC system Thermo Scientific Dionex Ultimate 3000 RSLC (Germering, Germany) consisting of a 6-channel degasser SRD-3600, a high-pressure gradient pump HPG-3400RS, autosampler WPS-3000TRS, and a column compartment TCC-3000RS equipped with a narrow-bore Hypersil GOLD™ C18 (2.1 × 50 mm, 1.9 μm) column (Thermo Fisher Scientific, Waltham, MA, USA)). Each chromatographic run was carried out isocratically with a mobile phase consisting of water–acetonitrile–methanol–acetic acid (25:50:25:0.2). The solvent flow rate was 300 μL min^−1^. Full MS—SIM was used as the MS experiment in negative and positive modes, where the resolution, automatic gain control (AGC) target, maximum injection time (IT), and mass range were 70,000 (at *m*/*z* 200), 3 × 106, 100 ms, and *m*/*z* 100–500, respectively. Xcalibur (Thermo Fisher Scientific, Waltham, MA, USA)) version 4.0 was used for data acquisition and processing.

### 3.1. Synthesis of 2-Amino-N-Phenethylbenzamide 3 and Its Diamides ***4a**–**d***

2-amino-*N*-phenethylbenzamide (**3**) [25,26,27]

To a solution of 3 mmol 2-amino-N-phenethylbenzamide **3**, 3.5 mmol of the corresponding acyl chloride in dichloromethane (10 mL) was added. Then, 3.4 mmol TEA was added after 10 min. After about 30 min, the reaction mixture was washed consequently with diluted HCl (1:4), Na_2_CO_3_, and H_2_O; dried with anhydrous Na_2_SO_4_; filtered on the short column filled with neutral Al_2_O_3_; and concentrated. Spectral data confirmed the structure of diamides **4a**–**d** (Supplementary Materials Figures S1–S12) and the literature data for **4b [31]**.

2-benzamido-*N*-phenethylbenzamide (**4a**): ^1^H-NMR (500 MHz, CDCl_3_) δ 12.06 (s, 1H, CONH), 8.73 (dd, J = 8.3, 4.4, 1H, Ar), 8.02 (d, J = 7.3, 2H, Ar), 7.49–7.55 (m, 3H, Ar), 7.43–7.47 (m, 1H, Ar), 7.29–7.34 (m, 3H, Ar), 7.20–7.25 (m, 3H, Ar), 6.98 (dd, J = 7.0, 1H, Ar), 6.55–6.58 (m, 1H, CONH), 3.69–3.73 (m, 2H, CH_2_), 2.94 (t, J = 7.1, 2H, CH_2_); ^13^C-NMR (126 MHz, CDCl_3_) δ 169.21, 165.67, 139.80, 138.61, 134.85, 132.57, 131.89, 131.44, 128.81, 128.81, 127.43, 126.54, 126.76, 122.88, 121.56, 120.71, 41.13, 35.53; FT-IR, cm^−1^: 3399 ν(-N-H, >NH-amide), 3063,3028 ν(Csp_2_-H, -Ph), 2925 ν_as_ (Csp^3^-H, -CH_2_), 2857 ν_s_(Csp^3^-H, -CH_2_), 1678, 1635 ν(>C=O), secondary amide I, 1596 ν(C-C=C, -Ph), ν(C-C=C, -Ph_mono_), 1526 δ(N-H), ν(C-N), trans-secondary amide II, 1495 ν(C-C=C, -Ph_ortho_), ν(C-C=C, -Ph_mono_), 1448 ν(C-C=C, -Ph_ortho_), δ_s_(-CH_2_-), 1437 ν(C-C=C, -Ph_ortho_), δ_s_(-CH_2_-), 1283 ν(C-C=C, -Ph_ortho_), ν(C-N), secondary amide III, 1190 δ(-Csp^2^-H, -Ph_ortho_), δ(-Csp^2^-H, -Ph_mono_), ρ(-CH_2_-)/ ν(N-C) in –NH-CH_2_; HRMS Electrospray ionization (ESI) *m*/*z* calcd for [M + H]^+^ C_22_H_21_O_2_N_2_^+^ = 345.15975, found 345.15891 (mass error ∆m = −2.45 ppm).

2-chloro-*N*-(2-(phenethylcarbamoyl)phenyl)benzamide (**4b**) [31].

*N*-phenethyl-2-(2-phenylacetamido)benzamide (**4c**): ^1^H-NMR (500 MHz, CDCl_3_) δ 11.03 (s, 1H, CONH), 8.52 (d, J = 7.8, 1H, Ar), 7.36–7.39 (m, 3H, Ar), 7.31–7.35 (m, 3H, Ar), 7.23–7.27 (m, 3H, Ar), 7.19–7.23 (m, 3H, Ar), 6.95 (td, J = 7.6, 1.5, 1H, Ar), 6.29 (broad s, 1H, CONH), 3.7 (s, 2H, CH_2_Bn), 3.62 (dd, J = 6.8, 2H, CH_2_), 2.87 (t, J = 7.1, 2H, CH_2_); ^13^C-NMR (126 MHz, CDCl_3_) δ 169.92, 168.84, 139.28, 138.65, 134.71, 132.37, 129.53, 129.46, 128.81, 127.22, 126.75, 126.30, 122.85, 121.49, 120.91, 49.14, 45.67, 41.16, 35.49; FT-IR, cm^−1^: 3337 ν(-N-H, >NH-amide), 3085, 3063, 3027 ν(Csp^2^-H, -Ph), 2935 ν_as_(Csp^3^-H, -CH_2_), 2863 ν_s_(Csp^3^-H, -CH_2_), 1685, 1631 ν(>C=O), secondary amide I, 1597 ν(C-C=C, -Ph_ortho_), ν(C-C=C, -Ph_mono_), 1554 ν(C-C=C, -Ph_ortho_), ν(C-C=C, -Ph_mono_), 1517 δ(N-H), ν(C-N), trans-secondary amide II, 1496 ν(C-C=C, -Ph_ortho_), ν(C-C=C, -Ph_mono_), 1446 ν(C-C=C, -Ph_ortho_), δ_s_(-CH_2_-), 1324 ν(C-C=C, -Ph_mono_), 1297 ν(C-C=C, -Ph_ortho_), ν(C-N), secondary amide III, 1159 δ(-Csp^2^-H, -Ph_ortho_), δ(-Csp^2^-H, -Ph_mono_), ρ(-CH_2_-)/ν(N-C) in –NH-CH_2_; HRMS Electrospray ionization (ESI) *m*/*z* calculated for [M + H]^+^ C_23_H_23_O_2_N_2_^+^ = 359.17540 found 359.17438 (mass error ∆m = −2.85 ppm).

2-(2-chloro-2-phenylacetamido)-*N*-phenethylbenzamide (**4d**): ^1^H-NMR (500 MHz, CDCl_3_) δ 12.03 (s, 1H, CONH), 8.54–8.55 (m, 1H, Ar), 7.59–7.62 (m, 2H, Ar), 7.42–7.47 (m, 2H, Ar), 7.38–7.41 (m, 2H, Ar), 7.35–7.37 (m, 2H, Ar), 7.32 (td, J= 7.1, 1.5, 1H, Ar), 7.29 (d, J = 3.4, 1H, Ar), 7.25–7.27 (m, 2H, Ar), 7.06 (td, J = 7.6, 1, 1H, Ar), 6.33 (broad s, 1H, CONH), 5.47 (s, 1H, CHCl), 3.74 (dd, J = 6.6, 3.3, 2H, CH_2_), 2.97 (t, J = 7.1, 2H, CH_2_); ^13^C-NMR (126 MHz, CDCl_3_) δ 168.62, 166.63, 138.65, 138.59, 136.94, 132.50, 129.10, 128.90, 128.84, 127.90, 126.79, 126.39, 123.66, 121.55, 121.40, 62.22, 41.18, 35.47; FT-IR, cm^−1^: 3300 ν(-N-H,-C(=O)-NH-C-), 3007 ν(Csp^2^-H, -Ph), 2924 ν_as_ (Csp^3^-H, -CH_2_-), 2854 ν_s_(Csp^3^-H, -CH_2_-), 1676 ν(>C=O), secondary amide I, 1628 ν(C-C=C, -Ph_mono_), 1597 ν(C-C=C, -Ph_ortho_), ν(C-C=C, -Ph_mono_), 1584 ν(C-C=C, -Ph_ortho_), ν(C-C=C, -Ph_mono_), 1514 ν(C-C=C, -Ph_ortho_), ν(C-C=C, -Ph_mono_), δ(N-H) and ν(C-N), trans-secondary amide II, 1447 ν(C-C=C, -Ph_ortho_), ν(C-C=C, -Ph_mono_), δ_s_(-CH_2_-), 1320 ν(C-C=C, -Ph_mono_), 1298 ν(C-C=C, -Ph_ortho_), 1228 δ(C-C=C, -Ph_ortho_), ν(C-N), secondary amide III, 1195 δ (C-C=C, -Ph_mono_), 1161 δ(C-C=C, -Ph_mono_), δ (-Csp^2^-H, -Ph_ortho_), ρ(-CH_2_-)/ν(N-C), 1109 δ(C-C=C, -Ph_mono_), δ (-Csp^2^-H, -Ph_ortho_), ρ(-CH_2_-)/ν(N-C), 1047 δ(-Csp^2^-H, -Ph_ortho_), δ(-Csp^2^-H, -Ph_mono_), 1030 δ(-Csp^2^-H, -Ph_ortho_), δ(-Csp^2^-H, -Ph_mono_), ν(C-N)/ρ(-CH_2_-); HRMS Electrospray ionization (ESI) *m*/*z* calculated for [M + H]^+^ C_23_H_22_O_2_N_2_Cl^+^ = 393.13643 found 393.13556 (mass error ∆m = −2.22 ppm).

### 3.2. In Silico Pharmacokinetic Profiling and Toxicity Analysis

The physicochemical and pharmacokinetic parameters, and drug-likeness of SQ were analyzed using the SwissADME free web tool [56].

The ProToxII web tool was used to predict acute and organ toxicity, toxicity class, and LD_50_ for the compounds [57,58].

A computer-based free web PASS Online program (Way2drug.com) was used to calculate the expected biological activities of the compound, based on its structural formula. Computational tools of the platform allowed for the prediction of several thousands of biological activity types, including the interaction with molecular targets, pharmacotherapeutic and side effects, metabolism, acute toxicity for rats, cytotoxicity, influence on gene expression, and other properties characterizing the evaluation of how promising the particular drug-like compounds were as potential pharmaceuticals [59,60,61,62,63].

### 3.3. In Vitro Inhibition of Albumin Denaturation

The inhibition of albumin denaturation was conducted using Milusheva’s method [60], with modifications.

### 3.4. SM Activity

#### 3.4.1. Solutions and Chemicals

The composition of the Krebs solution was as follows (in mM): NaCl 120, KCl 5.9, CaCl_2_ 2.5, MgCl_2_ 1.2, NaH_2_ PO_4_ 1.2, NaHCO_3_ 15.4, and glucose 11.5. The fluid was aerated with a mixture of 95% O_2_ and 5% CO_2_, and pH was maintained at 7.40.

The used drugs were acetylcholine (ACh), and mebeverine. All solvents, drugs, and reagents were purchased from Merck (Merck Bulgaria EAD).

Concentrated stock solutions of these substances were prepared in a solvent recommended by the manufacturer; aliquots of these stock solutions were added to the tissue baths to obtain the desired concentration. The volume of these aliquots was between 0.5 and 1% of the total volume of the bathing solution; pH was measured continuously with the pH meter HI5521 (Hanna Instruments Inc., Woonsocket, RI, USA). Deionized water with a conductivity of 18.2 mΩ/cm^2^ was used in all experiments.

#### 3.4.2. SM Preparations from Wistar Rats

Male Wistar rats with bodyweights in the range of 250–280 g (age 10–12 weeks) were used. All experimental procedures were performed in strict agreement with the current European regulations (86/609/EEC) regarding the protection of animals used for experimental purposes. The animals were housed under standard conditions, as follows: a temperature of 22 ± 2 °C, free access to food, and a 12 h light/dark cycle. Two or three muscle strips were taken from one rat gastric corpus in situ, separating the muscle tissue and preserving the mucosa intact. Circular dissected SMPs, 12–13 mm long and 1.0–1.1 mm wide, were used to record the CA isometrically. The preparations indicated by n were obtained under conditions of the continuous irrigation of tissues with a pre-aerated preparation solution containing NaCl/KCl/CaCl_2_ in a 27.2/1.1/1 ratio with a temperature of 4 °C.

All procedures were approved by the Institutional Animal Care Bulgaria and complied with the EU Directive 2010/63/EU.

#### 3.4.3. Mechanical Activity Registration of Rat Circular Gastric SMs

The mechanical activity of the SMPs was registered isometrically. The initial mechanical tension of the SMPs was achieved by stretching the tension system and had a value corresponding to a stretching force of 10 mN. The tone level of the preparations, after 60 min at stabilized spontaneous activity, was accepted as the initial tone. During the adaptation period, the Krebs solution was replaced 4 times. Alterations in the spontaneous mechanical activity and tone were recorded isometrically using a system of 15 mL organ baths (Tissue Organ Bath System 159920 Radnoti, Dublin, Ireland). The viability of the SMPs was periodically tested by applying 10^−6^ M ACh in the tissue baths.

### 3.5. Histology and Immunohistochemistry

The histology and immunohistochemistry experiments were tested as described in [38].

The morphometric analysis involved tissue slices of 5 μm in thickness, obtained from the circular and longitudinal layers of SM cells, as well as in the myenteric plexus of the stomach. The intensity of the immune reaction in the stomach was measured in arbitrary units (AU) on slices immunostained for IL-1β and nNOS. The measurements were performed using DP–Soft, version. 3.2 (Olympus, Tokyo, Japan). The average intensity of the pixels was recorded in arbitrary units in the range of 0–256 on microphotographs of the stomach, with 0 being black, and 256 being white. A minimum of 50 points were measured in the stomach at a magnification of ×400. All measurements involved five slices per animal and an examination of all cross-sections of the stomach.

### 3.6. Statistics

The data obtained were expressed as the mean ± standard error of the mean (SEM). The number of tissue preparations used in each experiment was indicated by n. Statistical differences in measuring SM activity were tested using Student’s t-test; a probability of less than 5% (*p* < 0.05) was considered significant. All statistical analyses were performed using the specialized software, SPSS, version 16.0 (SPSS Inc., Chicago, IL, USA).

The one-sample t-test and Wilcoxon test were used for immunohistochemical analysis. Quantitative data were analyzed using GraphPad Prism, version 8.0.1 (GraphPad Software, La Jolla, CA, USA). The asterisk indicates significant differences between groups—*** *p* < 0.001.

### 3.7. Ethics Statement

Animals used in the experiments were male Wistar rats. The experiments were approved by the Ethical Committee of the Bulgarian Food Agency,№ 229/09.04.2019 and were carried out following the guidelines of the European Directive 2010/63/EU. The animals were provided by the Animal House of Medical University Plovdiv, Bulgaria.

## 4. Conclusions

In conclusion, a series of 2-amino-*N*-phenethylbenzamides were synthesized for IBS treatment. In silico data suggested that these compounds could serve as potential orally active spasmolytics with minimal or no toxicity. This prediction was confirmed by ex vivo studies demonstrating their spasmolytic activity. Unlike mebeverine, which completely inhibited the ACh reaction, compounds **3** and **4a**–**d** preserved this response.

Furthermore, four of these compounds exhibited superior anti-inflammatory activity, as indicated by their in vitro ability to prevent albumin denaturation, surpassing acetylsalicylic acid and diclofenac sodium. Ex vivo tests evaluated the impact of the compounds on interleukin-1 (IL-1β) and nNOS expression, aiming to clarify the mechanism of anti-inflammatory action. Compounds **4b** and **4c** demonstrated enhanced anti-inflammatory activity by suppressing IL-1β expression in SM cells and neurons within the myenteric plexus while stimulating nNOS expression. Conversely, compounds **3** and **4a** lacked anti-inflammatory potential, as they stimulated IL-1β expression and suppressed nNOS expression.

These findings characterize the 2-amino-*N*-phenethylbenzamides as biologically active relaxants that could potentially prevent inflammatory diseases in the GI tract.

## Figures and Tables

**Table 1:** Synthesis of anthranilic acid diamides.

4	R	Yield, %	mp, °C
**a**	C_6_H_5_	80	94–95
**b**	2-Cl-C_6_H_4_	81	85–86
**c**	CH_2_-C_6_H_5_	79	76–77
**d**	CH(Cl)C_6_H_5_	80	161–164

**Table 2:** ADME parameters of the compounds **3** and **4a**–**d** representing drug-likeness.

Compound	MW, g/mol	XLOGP3	ESOL log S	Log Kp, cm/s	RB	BA Score	SA Score	LD_50_, mg/kg
**3**	240.30	2.52	−3.08	−5.98	5	0.55	1.45	1000
**4a**	344.41	4.54	−4.82	−5.18	8	0.55	2.31	2025
**4b**	378.85	5.17	−5.41	−4.94	8	0.55	2.41	2000
**4c**	358.43	4.48	−4.78	−5.31	9	0.55	2.49	600
**4d**	392.88	5.20	−5.43	−5.00	9	0.55	3.11	2025

MW: Molecular weight, XLOGP3: Octanol/water partition coefficient, ESOL LogS: Estimated aqueous solubility, Fraction Csp^3^: Ratio of sp^3^ hybridized carbons over the total carbon count, RB: Rotatable bonds, BA: Bioavailability, SA: Synthetic accessibility, predicted LD_50_—lethal dose to 50% of animals.

## Data Availability

The data presented in this study are available on request from thecorresponding author.

## Supplementary Materials

The following supporting information can be downloaded at: https://www.mdpi.com/article/10.3390/molecules29143375/s1, Figure S1: ^1^H-NMR spectrum of compound **4a**, Figure S2: ^13^C-NMR spectrum of compound **4a**, Figure S3: FT-IR spectrum of compound **4a**, Figure S4: Mass spectrum of **4a**, Figure S5: ^1^H-NMR spectrum of compound **4c**, Figure S6: ^13^C-NMR spectrum of compound **4c**, Figure S7: FT-IR spectrum of compound **4c**, Figure S8: Mass spectrum of **4c**, Figure S9: ^1^H-NMR spectrum of compound **4d**, Figure S10: ^13^C-NMR spectrum of compound **4d**, Figure S11: FT-IR spectrum of compound **4d**, Figure S12: Mass spectrum of **4d**.
