# Supplementary material for: 2-Amino-N-Phenethylbenzamides for Irritable Bowel Syndrome Treatment
Source: Molecules. 2024 Jul 18;29(14):3375. doi: 10.3390/molecules29143375 (PMC11280360; doi:10.3390/molecules29143375)

2-amino-*N*-phenethylbenzamides for irritable bowel syndrome treatment

Miglena Milusheva, Mihaela Stoyanova, Vera Gledacheva, Iliyana Stefanova, Mina Todorova, Mina Pencheva, Kirila Stojnova, Slava Tsoneva, Paraskev Nedialkov, and Stoyanka Nikolova

Table of Contents:

Figure S1: 1H-NMR spectrum of compound 4a, page 2

Figure S2: 13C-NMR spectrum of compound 4a, page 3

Figure S3: FT-IR spectrum of compound 4a, page 4

Figure S4: Mass spectrum of 4a, page 5

Figure S5: 1H-NMR spectrum of compound 4c, page 6

Figure S6: 13C-NMR spectrum of compound 4c, page 7

Figure S7: FT-IR spectrum of compound 4c, page 8

Figure S8: Mass spectrum of 4c, page 9

Figure S9: 1H-NMR spectrum of compound 4d, page 10

Figure S10: 13C-NMR spectrum of compound 4d, page 11

Figure S11: FT-IR spectrum of compound 4d, page 12

Figure S12: Mass spectrum of 4d, page 13

Figure S1: ^{1}H-NMR spectrum of compound **4a**

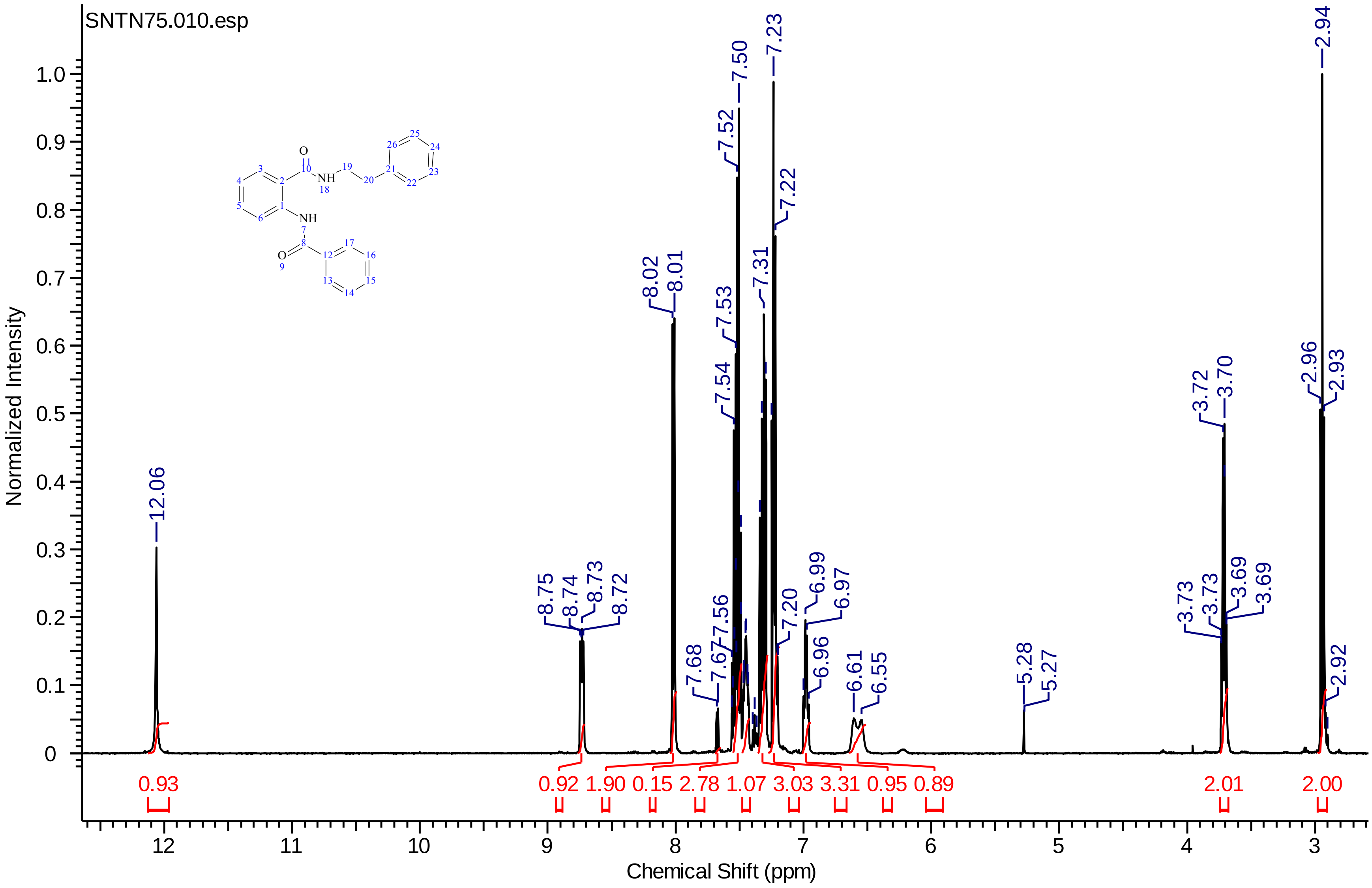

Figure S2: ^{13}C-NMR spectrum of compound **4a**

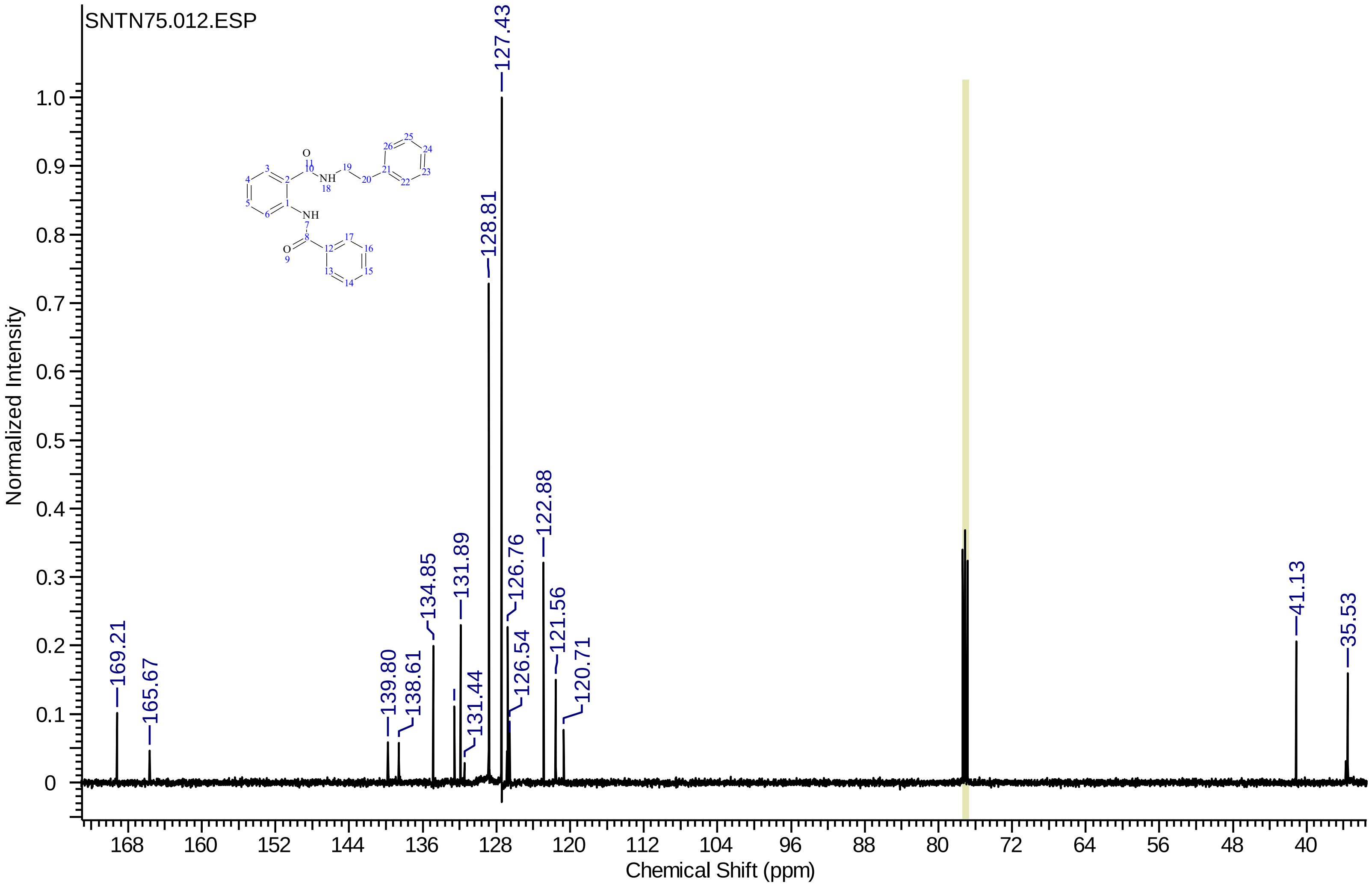

Figure S3: FT-IR spectrum of compound **4a**

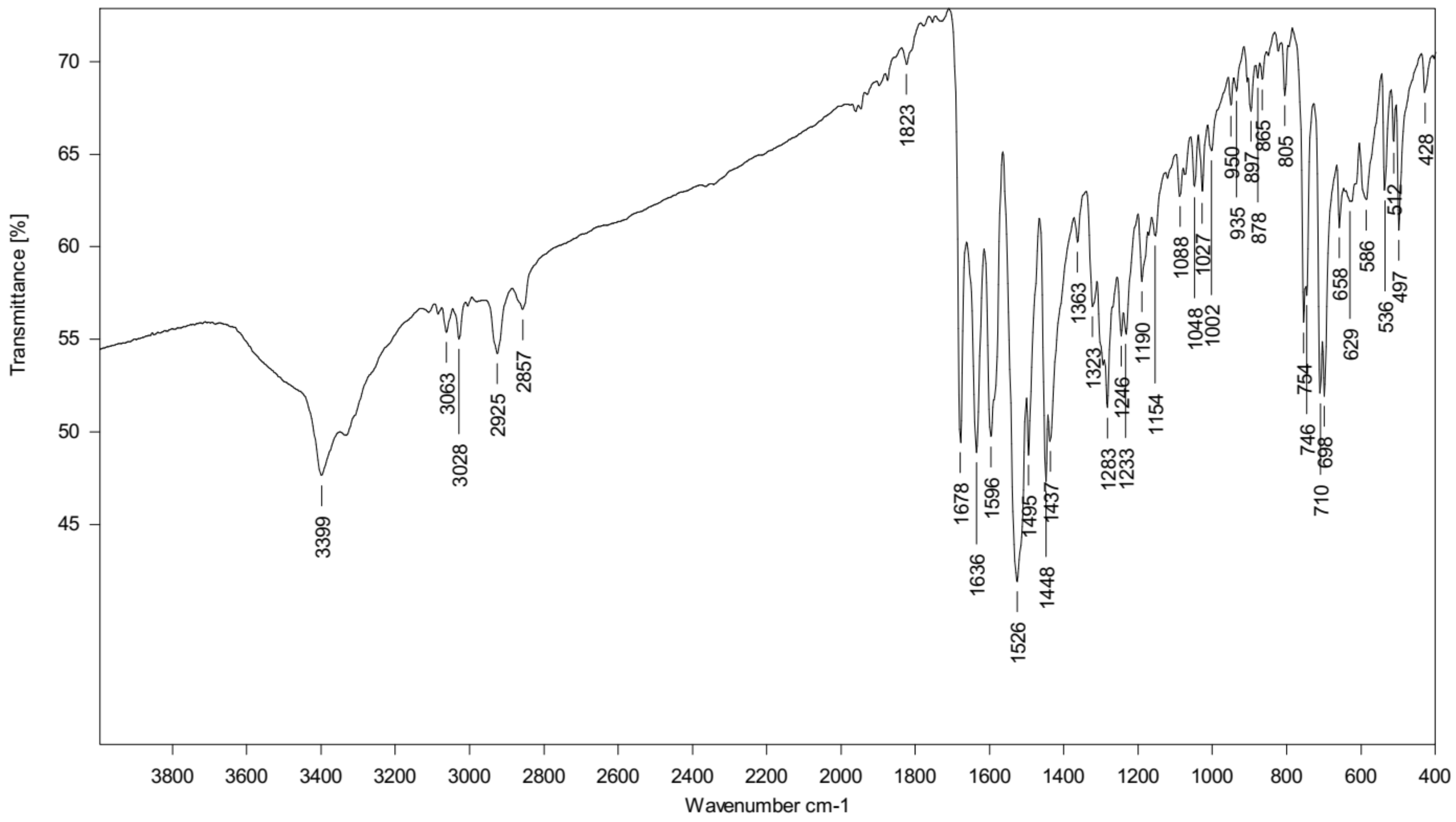

Figure S4: Mass spectrum of **4a**

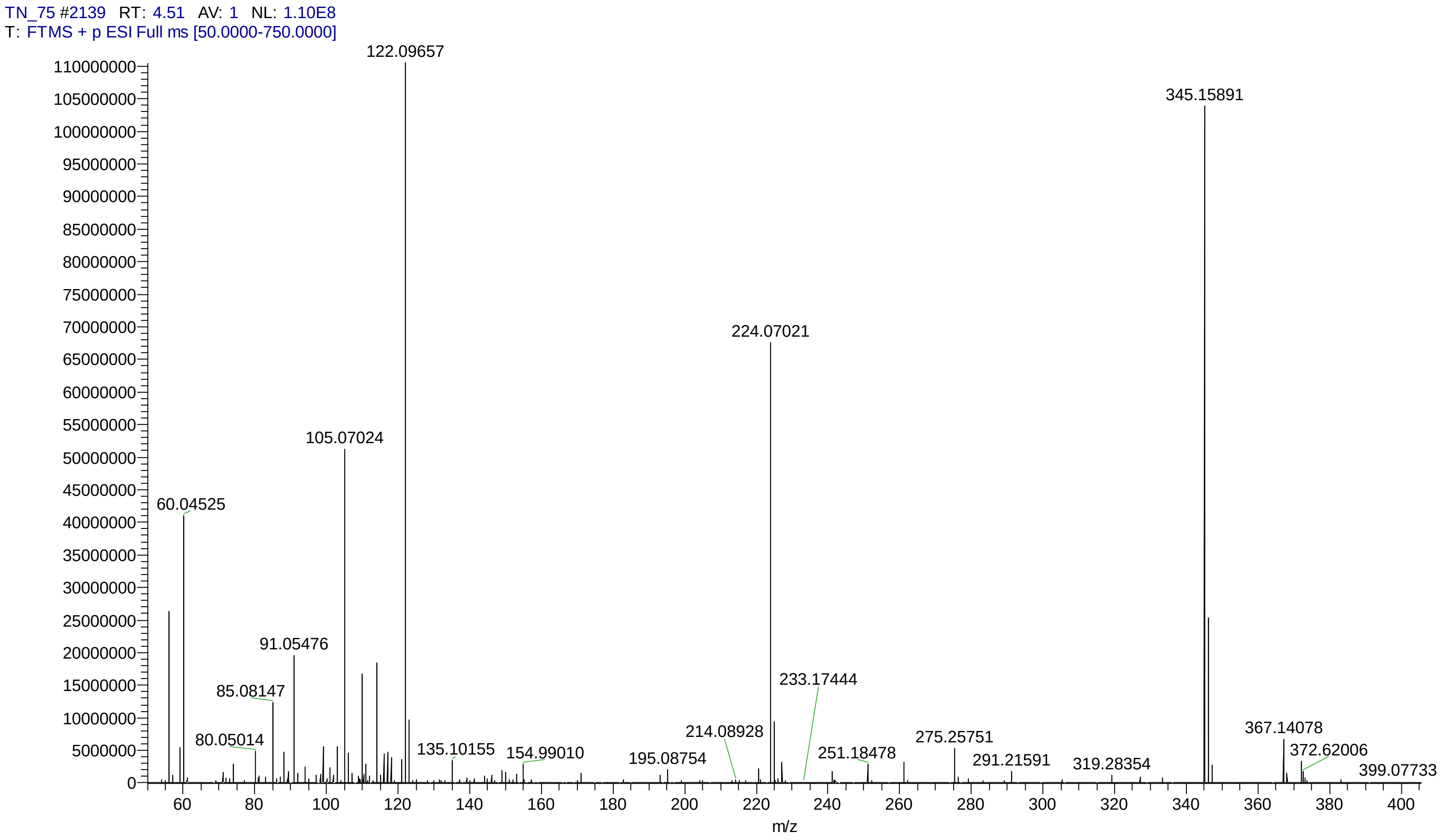

Figure S5: ^{1}H-NMR spectrum of compound **4c**

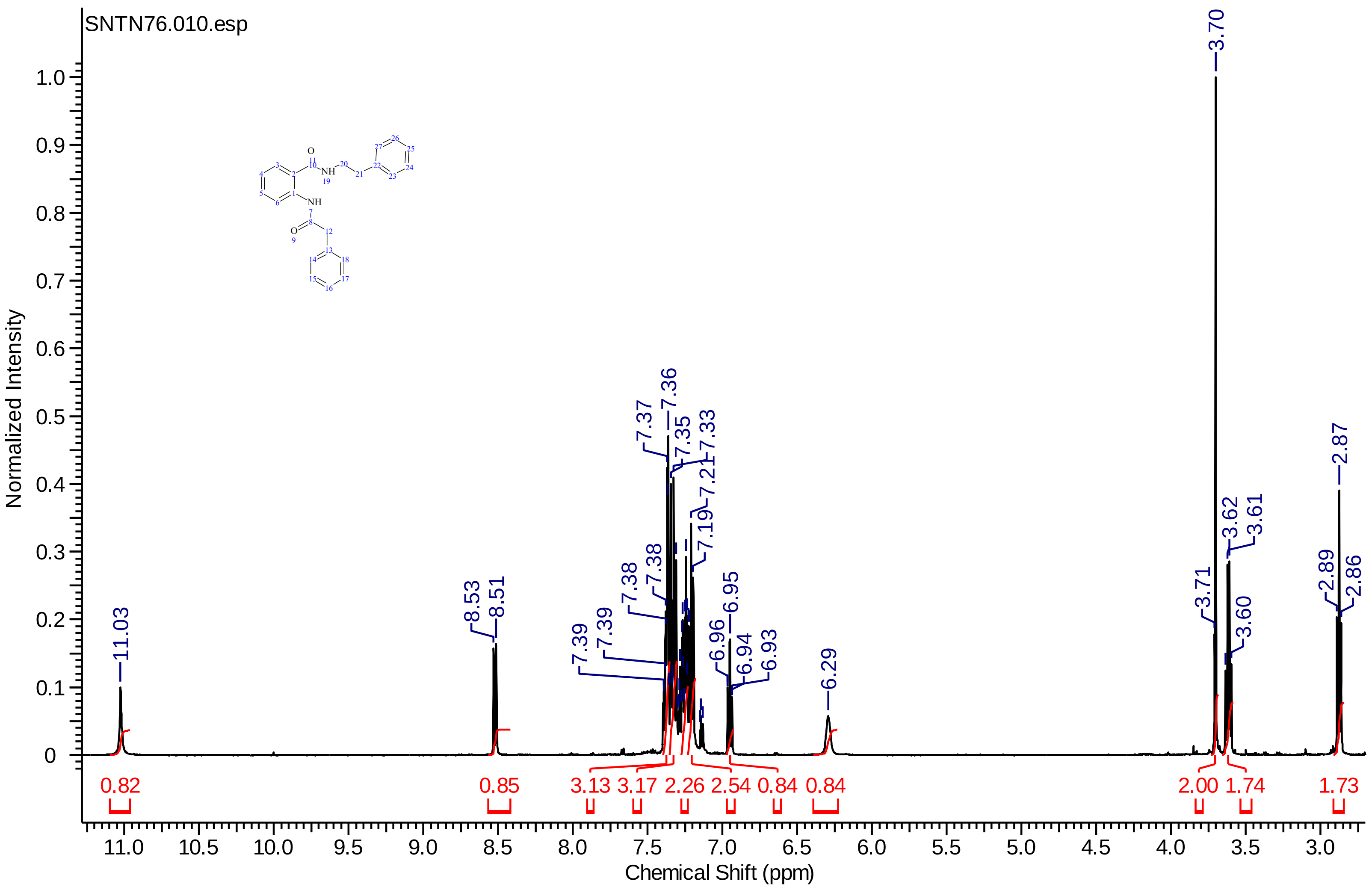

Figure S6: ^{13}C-NMR spectrum of compound **4c**

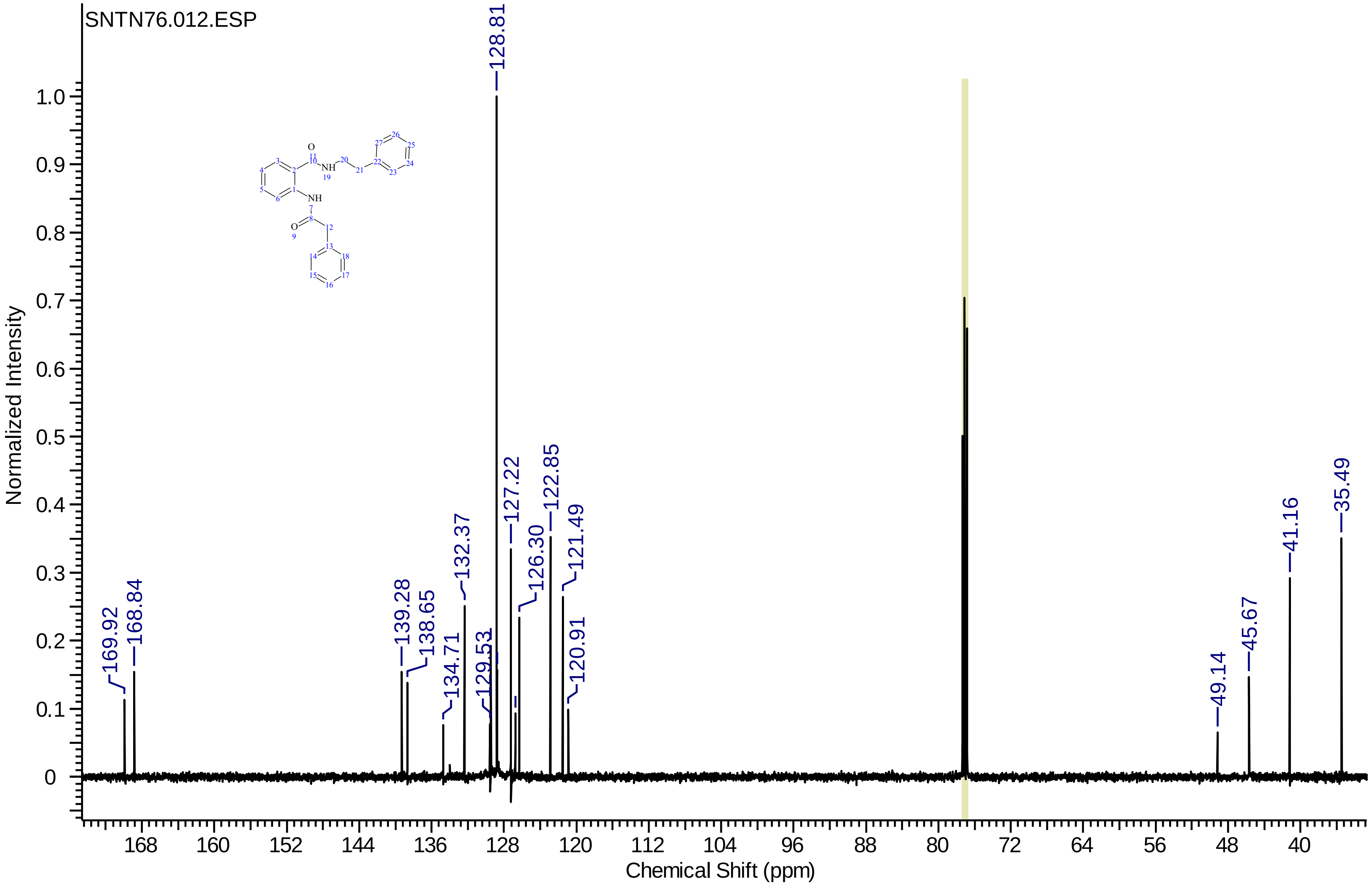

Figure S7: FT-IR spectrum of compound **4c**

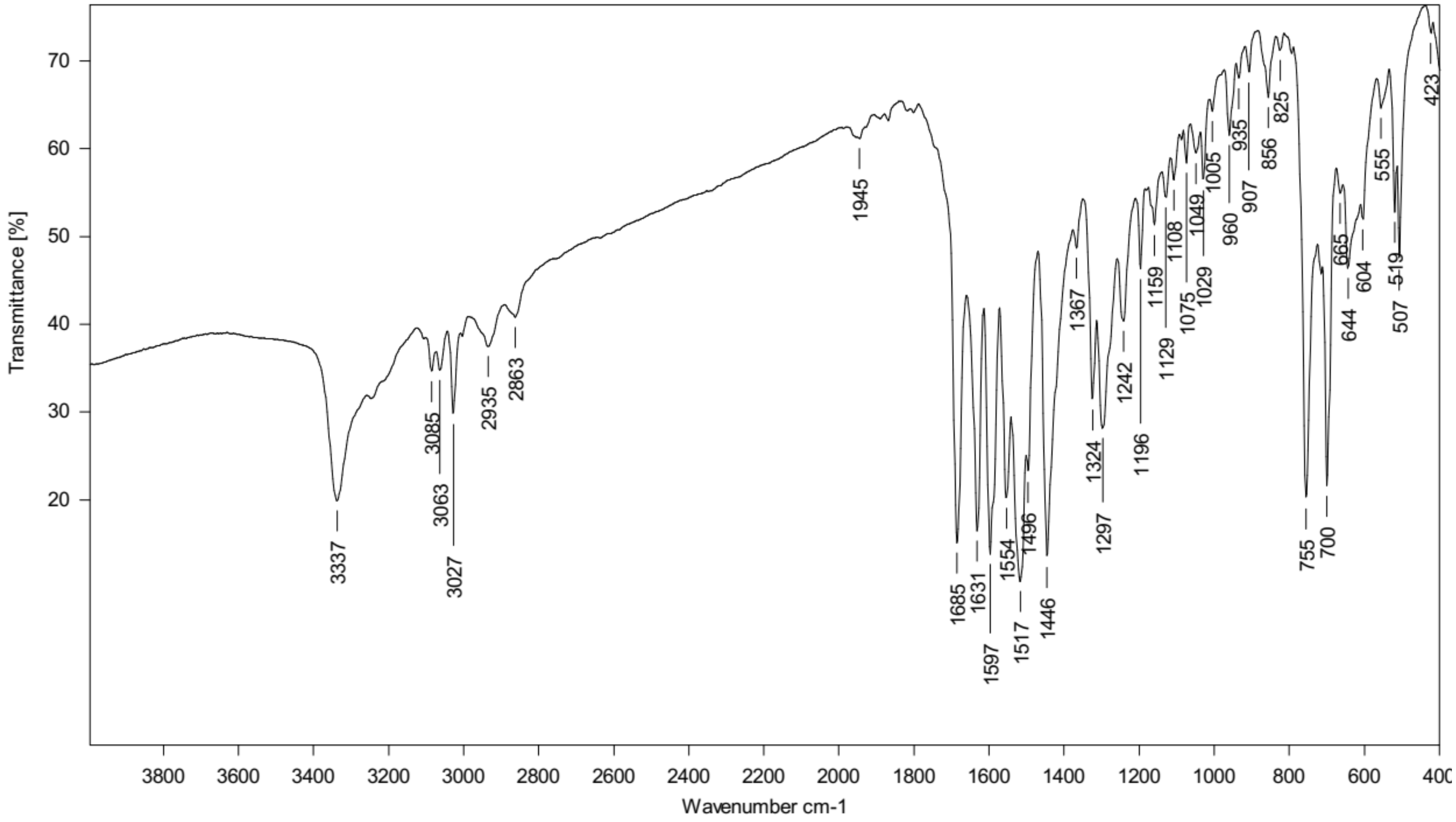

Figure S8: Mass spectrum of **4c**

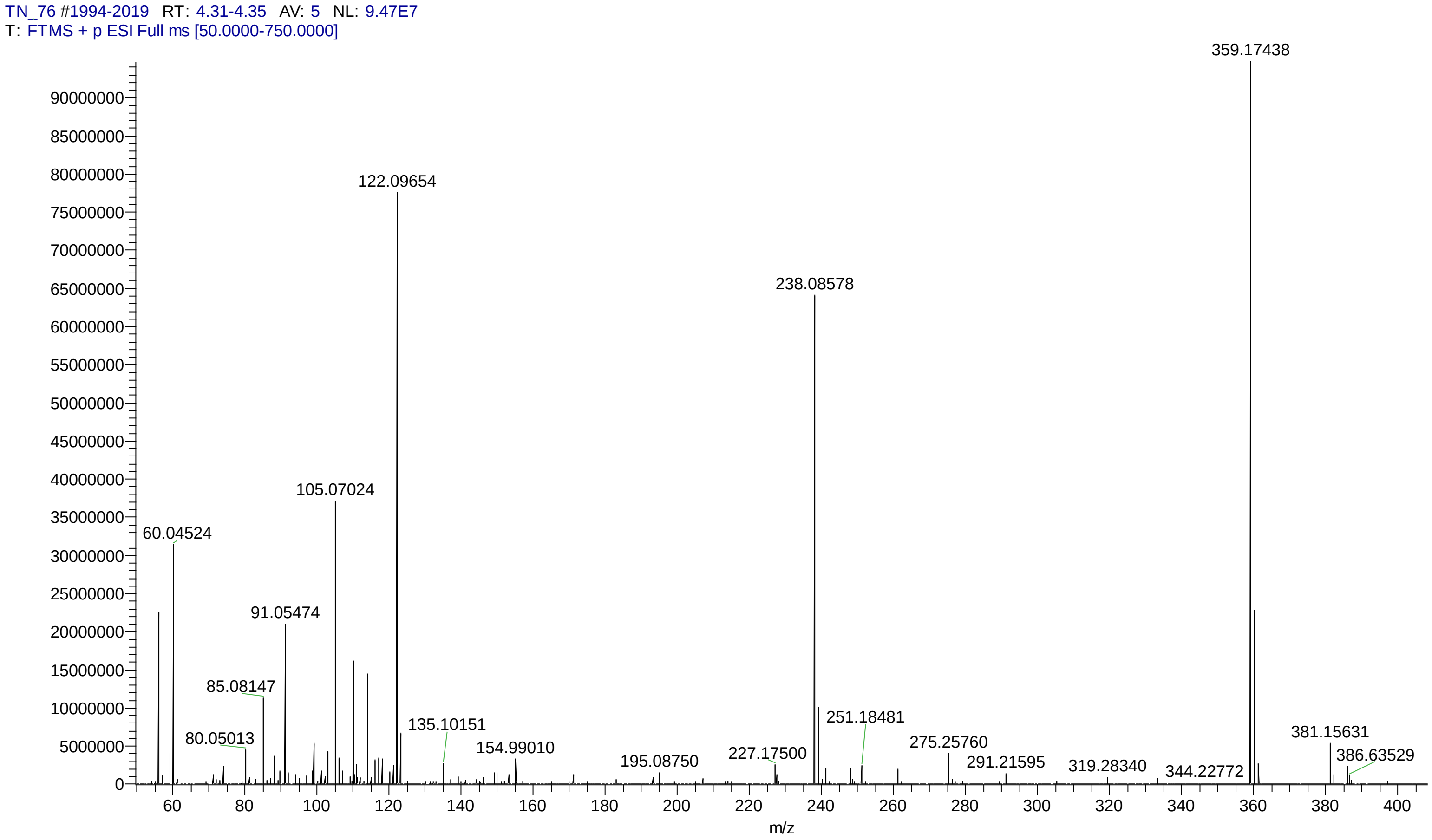

Figure S9: 1H-NMR spectrum of compound **4d**

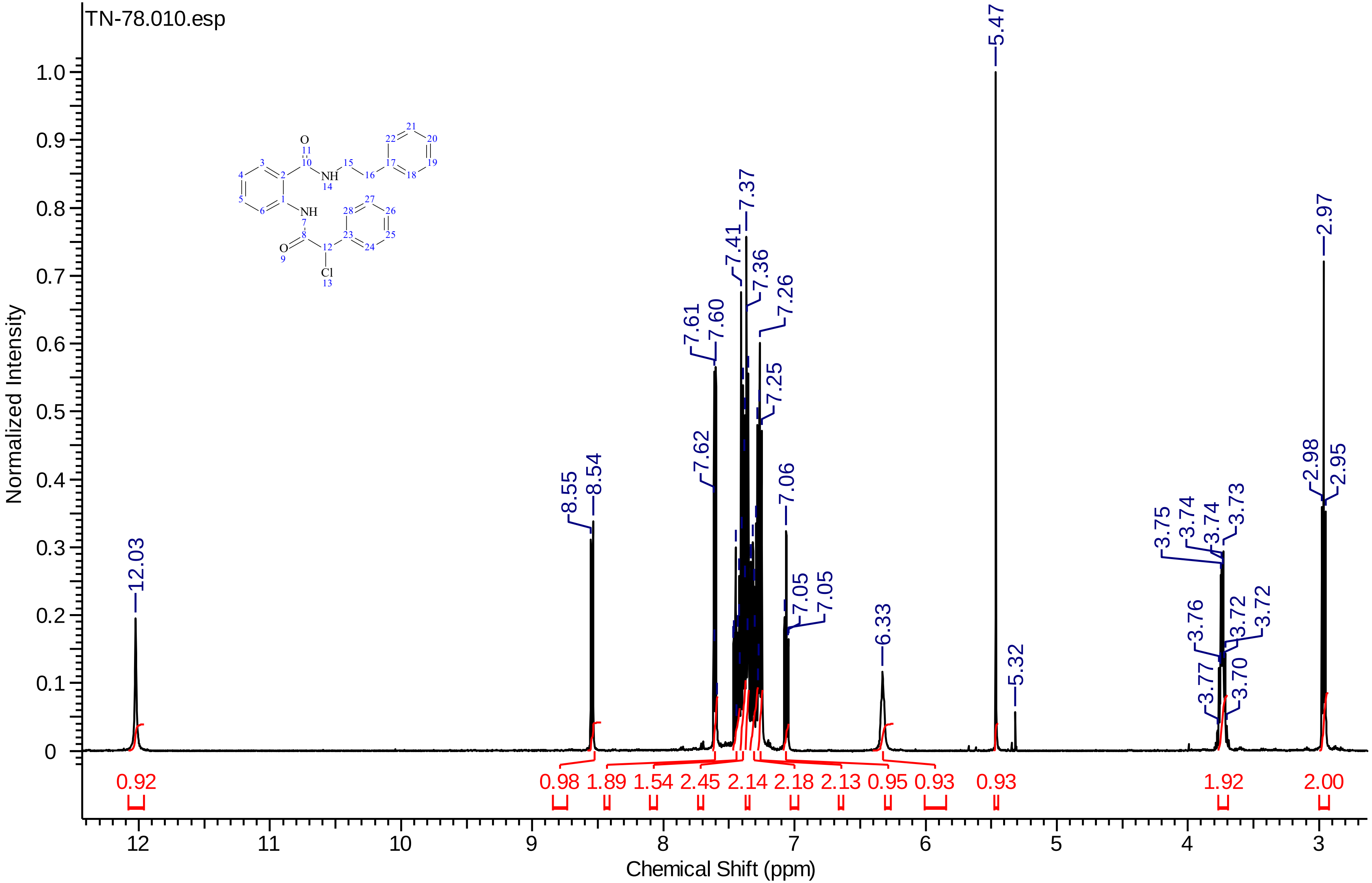

Figure S10: 13C-NMR spectrum of compound **4d**

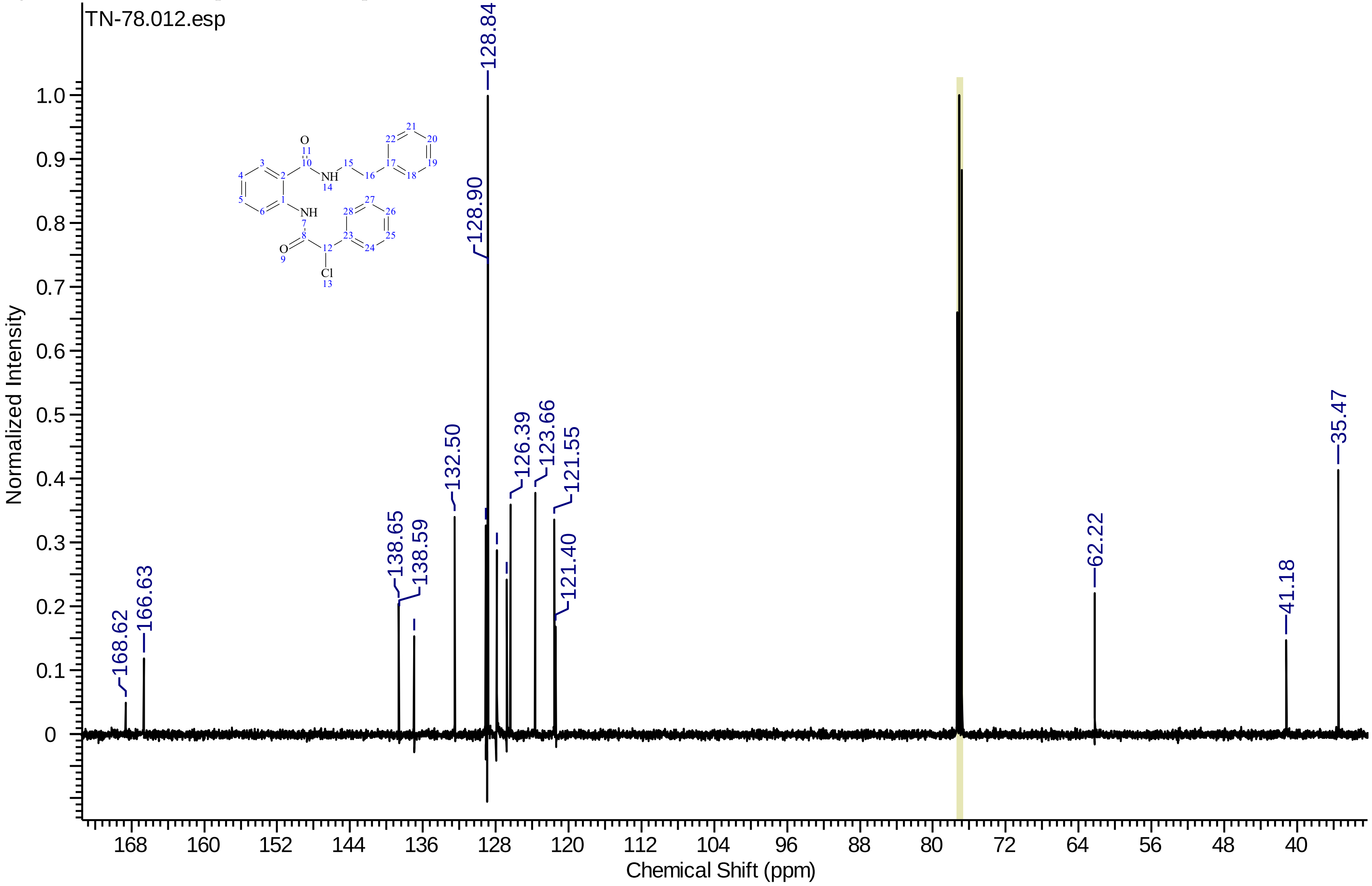

Figure S11: FT-IR spectrum of compound **4d**

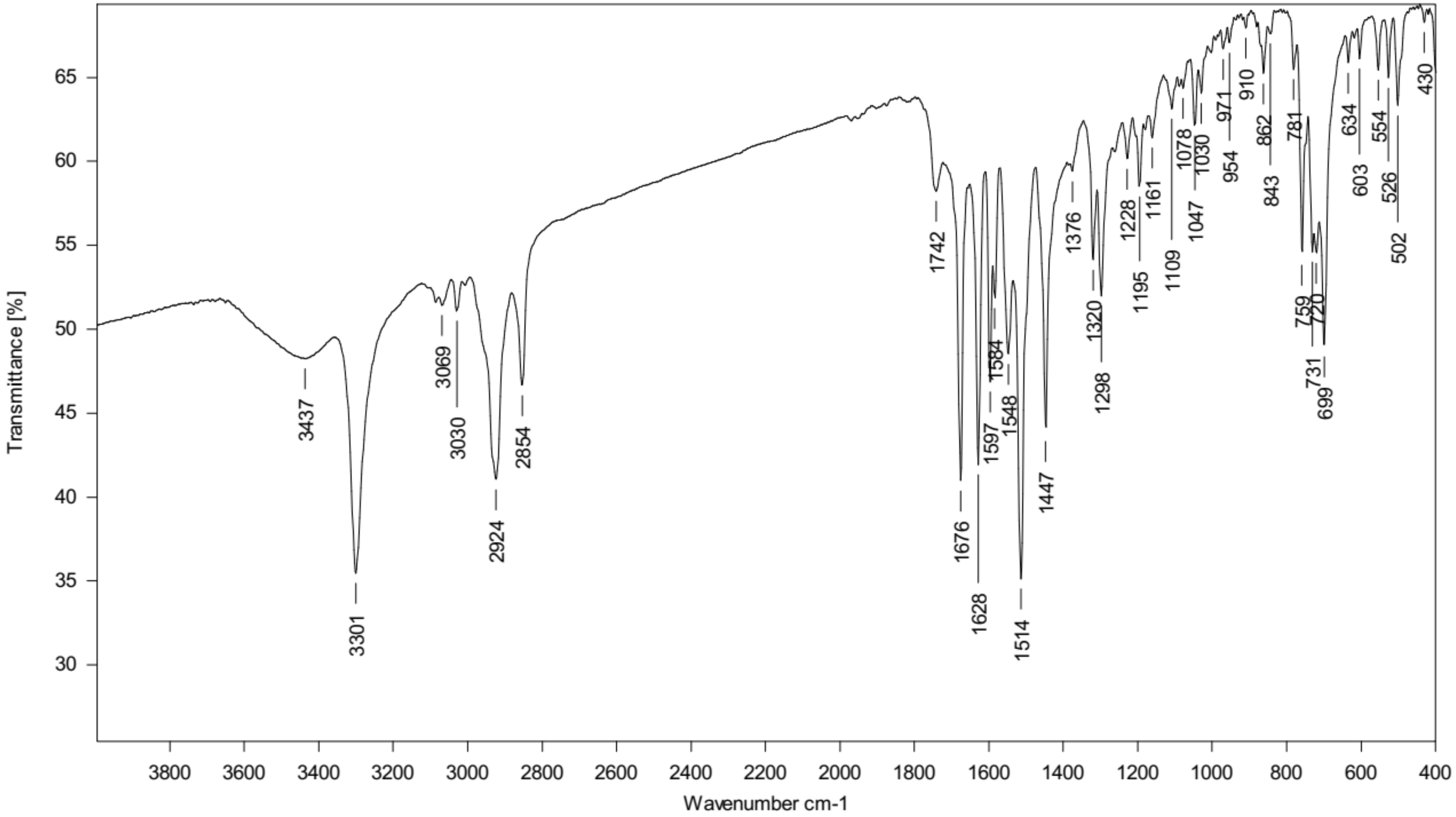

Figure S12: Mass spectrum of **4d**

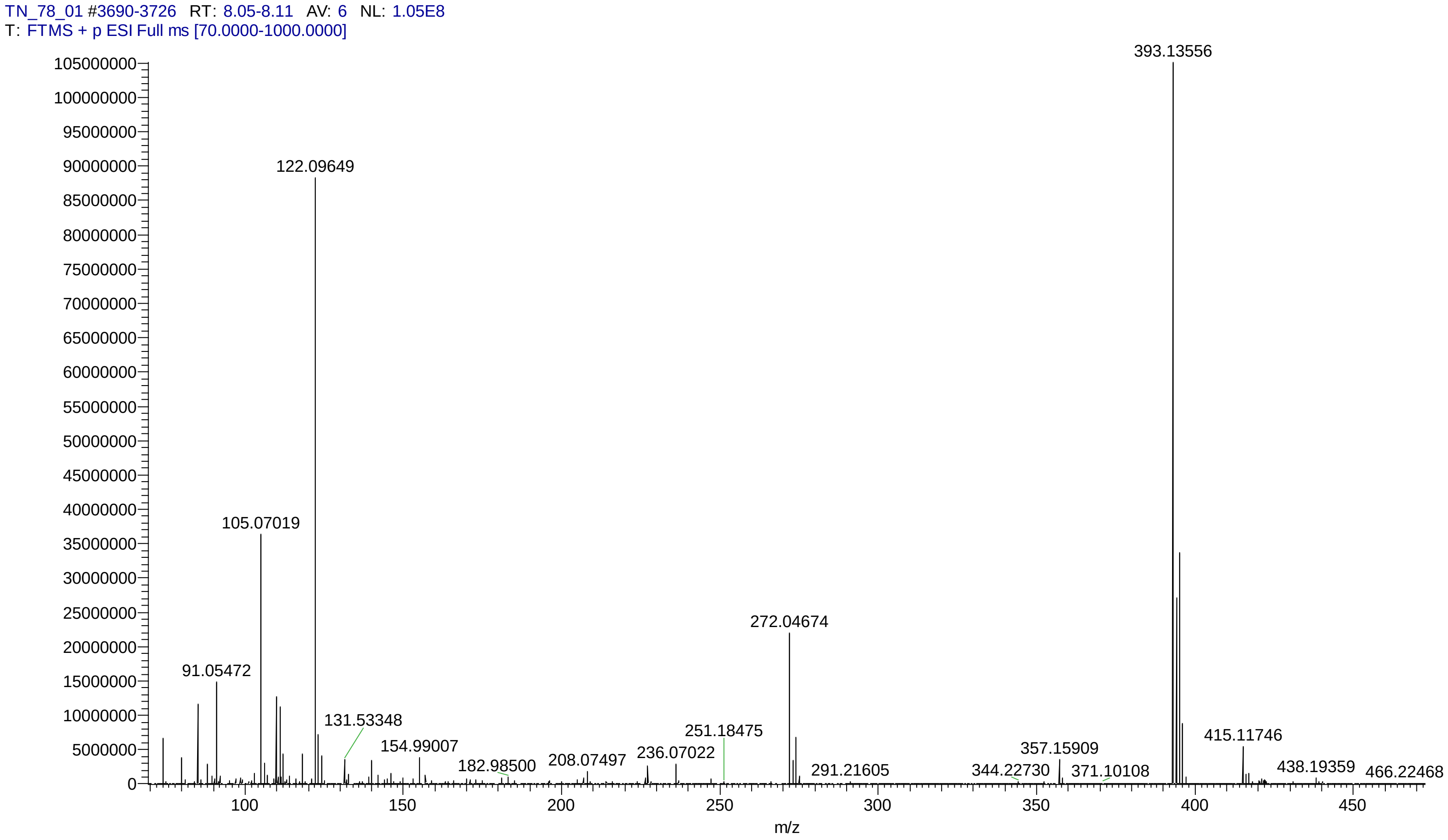

Supplement: Supplementary file 1 [file molecules-29-03375-s001.zip › molecules-3082944-supplementary.pdf]
